# A Review of Methods for Assessing the Environmental Health Impacts of an Agricultural System

**DOI:** 10.3390/ijerph15071315

**Published:** 2018-06-23

**Authors:** Leah Grout, Simon Hales, Nigel French, Michael G. Baker

**Affiliations:** 1 Department of Public Health, University of Otago, Wellington 6021, New Zealand; simon.hales@otago.ac.nz (S.H.); michael.baker@otago.ac.nz (M.G.B.); 2 Hopkirk Research Institute, Massey University, Palmerston North 4474, New Zealand; N.P.French@massey.ac.nz

**Keywords:** environmental health, impact assessment, agriculture, method review

## Abstract

(1) Background: Global agricultural production is projected to increase substantially in the coming decades. Agricultural production provides food and materials crucial to human survival and well-being and is a critical source of livelihood, providing employment opportunities and economic benefits. However, industrialized or intensified agricultural systems, in particular, can have adverse effects on public health, place pressure on natural resources, and reduce environmental sustainability. This review attempts to identify and characterize key environmental health assessment methods for examining a broad array of potential impacts; (2) Methods: Electronic databases Medline, Scopus, Web of Science, and GreenLINE were searched for published literature that presented methods for conducting an environmental health assessment of an agricultural system; (3) Results: Fifty-three sources were included in the review. Eight methods were selected to illustrate the wide range of approaches currently available: health risk assessment methods, health impact assessment, environmental impact assessment methods, environmental burden of disease, lifecycle methods, integrated assessment modeling, trade-off analysis, and economic assessment; (4) Conclusions: This review can provide guidance for selecting an existing method or for designing a new method for assessing the environmental health impacts of an agricultural system.

## 1. Introduction

The complexity of considering the health impacts of agricultural production systems has been articulated by the International Food Policy Research Institute:

“*The process of agricultural production and the outputs it generates can contribute to both good and poor health, among producers as well as the wider population [1]*.”

Agricultural systems provide food, fiber, and other materials necessary for human survival and good health [2]. They also present important income and employment opportunities [2], which are critical determinants of health [3]. However, agricultural systems can also pose public health risks, including occupational health hazards, foodborne illnesses, and infectious diseases [2]. For example, in livestock production, certain animals are associated with different zoonoses [2,4]. Furthermore, the consumption of certain agricultural products is associated with a number of diet-related chronic diseases [2,5]. Agriculture can also impact human health through intermediary processes and the influence of agriculture on health through the process of environmental change is evident [2,6]. In recent years, concerns have also been raised about the impacts of genetically modified or genetically engineered organisms in agriculture [7].

Agricultural systems can vary substantially by type of product, farming practices used, location, size, system ownership, and other factors [2]. While all farms have some impact on the environment and public health, the types and extent of impacts are likely to be different for different types of farms [8]. Local and subsistence agriculture generally involves small-scale mixing of crops and livestock and the majority of production is used to support farm households or local communities, with little surplus [9]. The smaller farms that typify local and subsistence agriculture are likely to have localized environmental health impacts and be more sustainable than large intensive or industrial farms [8]. Prevailing intensive or industrial agricultural methods, which typically depend on the use of off-farm inputs such as fossil fuels and agrichemicals [5,10], raise serious environmental concerns beyond the local area where such operations are located [5,11]. Potential environmental impacts from large industrial or intensive farming operations include the pollution of air, water, and soil; loss of biodiversity; and contribution to climate change [5,11]. Global agricultural production is predicted to increase substantially over the coming decades [12,13] and the broad array of potential environmental health risks that may accompany the rapid intensification of agricultural systems merit investigation.

There are a number of different methods and tools available for examining the environmental impacts of an agricultural system. Reviews of the different methods have identified a wide variety of approaches, such as environmental risk mapping, lifecycle assessment, environmental impact assessment, multi-agent system, linear programming, and agro-environmental indicators [14,15]. However, to the authors’ knowledge, the methods available for assessing the potential public health impacts associated with an agricultural system have not been reviewed.

This paper provides a review of eight methods for conducting an environmental health assessment of an agricultural system. The term *environmental health assessment* is used broadly in this review to include direct impacts on human health and well-being, as well as environmental and economic aspects that may indirectly influence public health. This review does not seek to identify methods that can be applied to specific environmental health hazards, such as those associated with genetically engineered organisms, but instead examines methods that can consider a broad array of impacts associated with agricultural systems. This review also does not intend to rank methods definitively but provides a characterization of method capabilities, strengths and limitations, and examples of their application. Reviewing the available methods helps to increase clarity and enhance understanding of specific methods and tools that may otherwise be inconsistently applied.

This research was also propelled by the observation that economic forces and markets tend to drive environmental changes, with potential consequences for public health. There is an expectation from the public that policy-makers and regulators will balance economic benefits with environmental sustainability and human health and well-being considerations. This paper aims to identify useful tools for policy-makers at all levels of government, as well as scientific researchers, industry leaders, and other stakeholders.

The aims are (i) to identify environmental health assessment methods that allow for an examination of a broad array of impacts within a complex political and economic context; and (ii) to determine the applicability of selected methods to an investigation of an agricultural system.

## 2. Materials and Methods

The electronic databases Medline, Scopus, Web of Science, and GreenLINE were searched for published literature that presented methods for conducting an assessment of the health and/or environmental impacts of an agricultural system. Literature consulted included methods literature and the methods-relevant sections of selected empirical research reports. Key search terms included: environmental impacts, health impacts, or ecological impacts; and agricultural production, animal husbandry, dairy farming, or livestock production; combined with assessment, measurement, quantification, evaluation, calculation, or analysis (Appendix A). The search terms reflect, in part, the interest of the study team in assessing the environmental health impacts associated with the intensification of the dairy sector in New Zealand (see Box 1). Articles in languages other than English were excluded. There were no restrictions on publication dates. The literature search was conducted between 11 and 12 July 2017.

Box 1 **The case of the New Zealand dairy sector.**

In the past few decades, New Zealand (NZ) has seen an economically driven increase in dairy production. Dairy cattle numbers increased from 5.2 million in 2007 to over 6.4 million in 2017 [16]. NZ is the world’s top dairy exporter [17,18] and accounts for approximately one-third of the global dairy trade [17]. However, little research has examined the potential environmental health effects of dairy farming in the country.

Dairying is associated with both positive and negative human health effects. One major benefit of dairying is the provision of nutrients. Dairy products are major sources of high-quality protein and bioavailable micronutrients (for example, calcium) [19]. Dairying is also crucial to NZ’s economy, accounting for approximately 25% of NZ’s merchandise export earnings [17,18]. Additionally, the NZ dairy sector provides numerous employment opportunities, supporting over 49,000 jobs [20]. Employment and income directly impact an individual’s socioeconomic status, and socioeconomic status is a critical determinant of health [3]. However, there are a number of potential adverse health impacts associated with dairy farming.

Environmental pollution from the dairy sector can have indirect impacts on human health and well-being. The global dairy sector contributes methane, nitrous oxide, and carbon dioxide to climate change [21,22,23,24,25], and particulate matter, nitrogen oxides, volatile organic compounds, and ammonia to air pollution [23,24]. Farm wastes can also generate odorants, including ammonia, hydrogen sulfide, and other compounds, which can impact local well-being [26]. Climate change can impact human health in a variety of ways, largely adversely [27,28,29] and air pollution is also a major, global environmental health risk that contributes to cardiovascular and respiratory disease morbidity and mortality [30].

Dairy cattle and other livestock can also have a major impact on water use and availability, water quality, hydrology, and the health of aquatic ecosystems [22]. Major sources of water pollution from dairy farms include animal wastes, which can carry zoonotic pathogens or high nutrient loads, pharmaceutical residues (for example, antibiotics, hormones), fertilizers and pesticides used for growing feed crops, and sediment from eroded pastures [22]. Antimicrobial-resistant pathogens or antibiotic resistant genes may also spread from farm animals into the surrounding environment with potential consequences for human health [31,32,33].

The dairy industry in NZ provides an excellent case-study for examining available assessment tools, as the intensification of dairy farming is a complex issue in which economic, environmental, health, and social goals are often at odds. Additionally, this example represents a challenging scenario where more information is needed about the impacts of a rapidly changing, complex system.

After searching each database, individual article titles were assessed to determine their relevance to the topic of this review. Articles that did not focus on human or environmental health impacts were excluded. Additionally, articles that examined multiple impacts were preferentially selected over those that focused on measuring or surveying a single potential hazard. Several environmental health assessment methods were identified by the study team prior to the search and the additional methods were identified from the literature search. A wide variety of methods were selected to analyze the wide diversity of approaches, but this was not intended to be an exhaustive review.

In the next stage of the study, selected empirical research reports were categorized according to the method used. Articles selected from the methods literature were also categorized by the method(s) or methodological family discussed. Each discrete method was then described according to key characteristics, including the development and intended aims. The purpose, general process, included dimensions (economic, environmental, health, social), and strengths and limitations for the selected methods were considered with regards to the suitability and utility of each approach for assessing the potential impacts of an agricultural system. A number of other factors were also broadly considered (reference Table 3 in Section 4):

- Types of decisions supported: (a) assessing the impacts of products or processes; (b) assessing policies, programs, projects, or plans
- Temporal scale: short-term impacts (for example, weeks, months), long-term impacts (for example, years)
- Spatial scale: local, regional, national, international/global
- Other considerations: timeframe for conducting an analysis; ease of use (for example, data requirements and technical expertise); management of uncertainty

In the final stage, examples of the past use of each method for the assessment of agricultural systems were briefly reviewed and the potential applicability of the methods to an assessment of the New Zealand dairy sector was discussed (reference Box 2 in Section 5).

## 3. Article Selection and Identification of Assessment Methods

**Figure 1:**
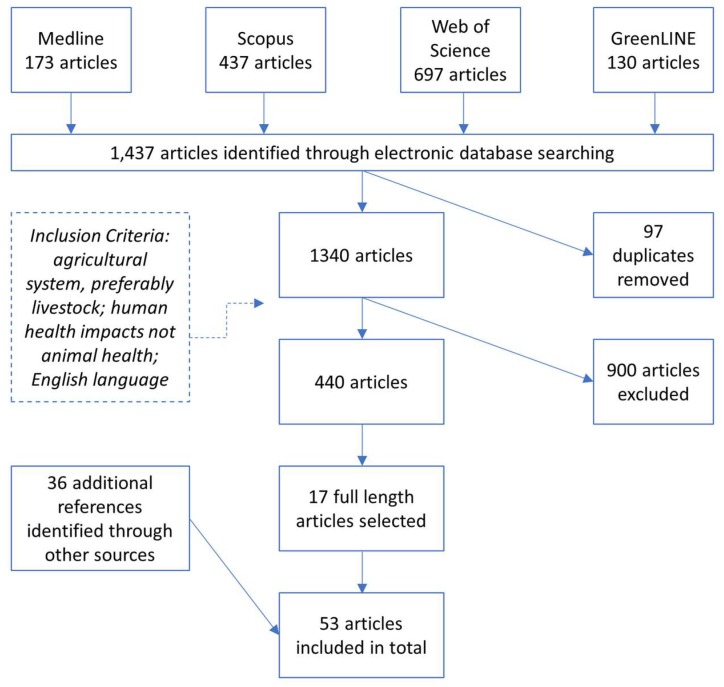
The search strategy and article selection flow diagram.

A total of 1437 papers were identified in the initial search (Figure 1). Ninety-seven duplicates were removed. Following the screening of articles by title, 444 eligible articles remained. After the application of the selection criteria, seventeen full-length articles were selected for inclusion in the review and an additional 36 reports and papers were identified through forward and back citation searching. In total, 53 articles, reports, and resources were included in the final review. While most of the methods included in the review were identified through the literature search, the environmental burden of disease approach was familiar to the study team and some literature related to the approach was identified externally to the electronic database search.

A review of the methods for assessing the environmental health impacts of an agricultural system reveals a diverse array of approaches. The approaches identified in the selected articles and reports could be classified into eight discrete methods for conducting an environmental health assessment: health risk assessment (HRA), health impact assessment (HIA), environmental impact assessment (EIA), the environmental burden of disease (EBD) approach, lifecycle assessment (LCA), integrated assessment modeling (IAM), trade-off analysis (TOA), and economic assessment (EA) (Table 1). While the approaches are broadly categorized into eight groups, cumulative risk assessment (CRA), which was developed out of the HRA method is also included in this review as a method sub-type. Similarly, environmental health impact assessment (EHIA) and strategic environmental assessment (SEA), which are methods that developed out of the EIA method, and lifecycle costing (LCC), which was developed out of the LCA method, are also included as a method sub-types.

## 4. Description of Methods

While this review was not intended to be exhaustive, the inclusion of eight discrete methods provides coverage of a wide range of approaches and showcases the diversity of available methods (Table 2). This review specifically focused on methods for the assessment of the environmental health impacts of an agricultural system. However, a number of the methods outlined here can also be used to assess economic and social factors associated with agricultural production. Many of these factors are important determinants of health in their own right [3]. Furthermore, in some cases, it is possible to combine a number of different methods or tools in order to provide a more holistic view of an agricultural system. For example, economic methods and tools have frequently been integrated with other approaches.

In this section, an overview of each selected method is presented (following the order shown in Table 1 and Table 2). However, the definitions given for each method in this review may not be universally accepted as they are based on a relatively small number of examples. Key method attributes, including the dimensions covered, primary end users, spatial and temporal scale, time to complete, ease of use, and consideration of uncertainty are briefly compared (Table 3).

### 4.1. Risk Assessment Methods

#### 4.1.1. Health Risk Assessment

Health risk assessment (HRA), which may also be referred to as environmental health risk assessment, is the process used to estimate the probability of adverse health effects in humans who may be exposed to a hazard [34]. HRA studies typically follow several different steps, including hazard identification and characterization, dose-response assessment, exposure assessment, risk estimation or characterization, and risk communication [4,34]. This basic framework is commonly used for conducting the quantitative aspects of other approaches such as HIA and EBD [44,56]. HRA allows for the analysis of health hazards in an objective and quantifiable way, and helps to determine where to intervene, how to allocate funds for risk control activities, and can identify knowledge gaps [4]. However, HRA cannot identify hazards or predict the emergence of new hazards; therefore, this methodology is only useful if the hazard is identified correctly and if the model, parameters, and data are all appropriately selected [4]. Furthermore, HRA cannot explicitly state how to respond to a health risk (although that function is typically part of subsequent risk management efforts), but it can indicate where to intervene [4]. The approach is also limited in that it typically only examines one health hazard at a time [4] and it does not include environmental, social, or economic outcomes.

#### 4.1.2. Cumulative Risk Assessment

Cumulative risk assessment (CRA) was developed out of the HRA approach when the United States Environmental Protection Agency (EPA) was directed by the Food Quality Protection Act (FQPA) of 1996 (although relevant activity had occurred prior to the FQPA mandate [40,42]) to examine the cumulative effects of chemical exposures that occur simultaneously, rather than only conducting single chemical assessments [40,42]. The FQPA specifically required the EPA in its assessment of pesticide safety to look at the cumulative effects of pesticides with common mechanisms of toxicity, considering the aggregate effects of multiple exposure pathways [40,42]. The EPA Science Policy Council (SPC) took the first step towards developing guidelines for the CRA approach when it issued guidance on planning and scoping for CRA in 1997 [40]. Then, in the early 2000s, the SPC tasked the Risk Assessment Forum with beginning an EPA-wide framework for the CRA process, further cementing the use of the approach in the United States [40].

The EPA’s framework provides a flexible structure for conducting a CRA that can differ from the traditional HRA approach in several ways: the CRA approach focuses on the combined effects of more than one agent or stressor, has an increased focus on the specific subpopulations at risk, and may include a wider variety of nonchemical agents or stressors [40,41,42]. Additionally, CRA is not always quantitative; it can also be qualitative [40,42]. Typically, a CRA study follows three main phases: (i) planning, scoping, and problem formulation; (ii) analysis; and (iii) risk characterization [40]. In the first phase, a team of experts and stakeholders establish the goals, scope, and focus of the assessment [40,42]. In the analysis phase, profiles of exposure are developed, interactions among agents and stressors are considered, and the risk to the population and subpopulations are assessed [40]. In the final phase, the risk estimates calculated during the analysis phase are interpreted; the estimates are put into perspective in terms of their significance, reliability, and the overall confidence in the assessment [40].

CRA studies can be conducted retrospectively to assess past or current risks or prospectively to determine the potential risks for proposals and projects [40]. This versatility allows CRA results to be used for meeting regulatory mandates; identifying targets for enforcement actions; informing policy, regulation, and permitting decisions; and for general education purposes [40]. Theoretically, the CRA approach may be better suited to real-world circumstances than the HRA approach [40]. However, the approach also carries with it certain challenges and many CRA studies have failed to follow the EPA guidelines [42]. CRA studies can be highly complex and require significant expense, effort, and time to complete, even with a narrow scope [40]. Additionally, CRA studies often need to combine many different types of data, which can add to the overall technical requirements and complexity [40]. Furthermore, the CRA approach cannot always incorporate social and economic dimensions [41].

### 4.2. Health Impact Assessment

Health impact assessment (HIA) is a predictive tool used to judge the actual or potential impacts of a policy, program, or project on public health and the distribution of potential impacts within the population [46,47,48]. An HIA study is usually a multidisciplinary process that utilizes a structured framework to examine a range of evidence about potential health impacts [46]. There is no fixed method for conducting an HIA study, but it typically follows the distinct screening, scoping, and appraisal stages [46,48] and often draws upon the HRA approach to quantify specific exposures [44]. HIA is most commonly applied to decisions made outside of the health sector and focuses on multiple determinants and dimensions of health, including a range of socioeconomic impacts [48]. The ultimate goal of an HIA study is typically to create evidence-based recommendations that minimize negative health effects and maximize health benefits [46,47,48].

HIA has developed in two distinct contexts [48]. First, governments and international institutions have led efforts to incorporate health considerations more broadly into the environmental impact assessment (EIA) approach (discussed below) [48]. The HIA approach and framework have been directly derived from EIA, but HIA was developed specifically because the EIA framework does not focus on health outcomes [47]. EIA commonly uses a biophysical health model which mainly focuses on environmental determinants like air quality and water pollution, whereas HIA attempts to incorporate the social determinants of health [47]. Second, the use of HIA has grown as part of efforts to include health goals more explicitly in social policy and urban planning [48]. HIA provides a promising framework to incorporate health-based design principles into land use planning decisions [48].

Key issues for the implementation of HIA relate to funding and training [46,48]. Assessments of larger and more complex proposals can require significant time, funding, and technical expertise [46,48]. Some of the other challenges involved in conducting an HIA include questions about the timing; if an HIA is attempted at too early a stage in the development of a proposal, then the policies or projects may still be too vague to allow for a strong assessment [46]. Conversely, if an HIA is conducted too late it will have limited ability to affect change and influence decisions [46]. Another issue for intersectoral HIA studies is that the causal pathways for potential impacts can be very complex and the current evidence base may not be strong enough to assess specific policy options [46]. However, one of the major strengths of HIA is that it can include health, environmental, social, and economic dimensions and can facilitate the consideration of public health across policy sectors [46]. Additionally, HIA benefits from stakeholder involvement, which allows those who might experience potential impacts to provide feedback and insight [46].

### 4.3. Environmental Impact Assessment Methods

#### 4.3.1. Environmental Impact Assessment

Environmental impact assessment (EIA) was established in the United States in 1969 but has since been used around the world [52]. EIA has been used to explore the effects of policies, programs, and projects on the environment and, in many countries, there are statutory requirements that EIA be undertaken for new policies or projects [53]. However, EIA policies and legislation have excluded agricultural sectors in many countries, despite the fact that agricultural projects and development have direct impacts on the environment [52]. EIA can be used as a predictive tool and allows for the identification and assessment of the environmental and socioeconomic effects of policies or projects [52]. However, EIA does not typically include an assessment of the potential health impacts of a proposal and those EIA studies that have included a health component tend to be narrowly focused [53]. Engagement with stakeholders is considered a critical part of the EIA process and can increase public awareness, help to correct misconceptions, and inform decision-makers about potential or perceived impacts [52]. EIAs generally seek to minimize environmental damage and guide sustainable development [50,52]. However, EIAs that are required by governments can be considered inconvenient by companies, producers, or stakeholders that would prefer to avoid the additional administrative costs associated with conducting an assessment [50,52].

#### 4.3.2. Environmental Health Impact Assessment

An environmental health impact assessment (EHIA) is an EIA that includes an assessment of health impacts in addition to environmental impacts [53]. The analysis of health issues is typically not as comprehensive as in an HIA, with health impacts included only as a single component in the assessment [53]. Additionally, health impact analysis within an EHIA may only include health impacts that are easily quantified, such as chemical exposures [53].

#### 4.3.3. Strategic Environmental Assessment

A strategic environmental assessment (SEA) is typically undertaken earlier in the decision-making process for proposals than EIA or EHIA [53]. SEA, as defined by European Directive 42/EC/2001 and the United Nations’ Economic Commission for Europe (UNECE) protocol, must place an emphasis on human health [54], and should include thorough consideration of health impacts in addition to environmental impacts. A review of SEA studies found that many considered health impacts related to natural or physical factors, but social and behavioral aspects were rarely included [54]. Generally, SEA provides a framework that could allow for health impacts to be fully considered within an environmental assessment [53].

### 4.4. Environmental Burden of Disease

The environmental burden of disease (EBD) approach assesses the disease burden attributable to environmental risk factors [56]. While quantitative environmental health assessment studies have traditionally focused on single risk factors, the EBD approach allows for the assessment of the health impacts of multiple different environmental risk factors and for the analysis of different scenarios of environmental change [56]. The evaluation of the disease burden of a risk factor requires the estimation of the harmful effects of that risk factor on human health and the distribution of harmful effects in the study population [56]. Generally, it is recommended that EBD studies use a causal web for the comparative quantification of health risks [55]. Causal webs are models that link distal and proximal risk factors in a causal inference cascade [55]. Distal and proximal causes may interact with each other and with health outcomes and a causal web allows for the assignment of mathematical functions to individual links [55]. The results of burden of disease studies are usually measured in terms of deaths and disability-adjusted life years (DALYs) [56].

Two general approaches can be used to assess the environmental burden of disease: an exposure-based approach and an outcome-based approach [55]. The exposure-based approach estimates the disease burden based on the distribution of an exposure in the population and is calculated by combining population exposures with appropriate dose-response relationships [55], similar to HRA. The outcome-based approach is based on the fraction of disease burden that is attributable to a specific risk factor [55]. The outcome-based approach calculates the disease burden by combining an attributable fraction with the disease burden of a specific health outcome [55]. Both approaches share the same underlying assumptions with regards to the links between health and the environment [55]. However, diseases that are primarily associated with a single risk factor are best suited to assessment using the outcome-based approach, while risk factors that can result in multiple diverse health outcomes are usually better suited to assessment using the exposure-based approach [55].

The EBD approach carries with it certain challenges. First, it is impossible to include all aspects of risk in an EBD study [56]. Second, complex causal pathways may have to be simplified for calculation and, in many cases, the data supporting environmental health links are of variable quality [55,56]. Some EBD studies do not attempt to model the complex interactions between environmental health factors (for example, the impacts of exposure to two pollutants may be higher when both are present together than when exposure occurs separately) and treat pollutants individually rather than considering joint effects [56]. Another challenge associated with linking environmental exposures to health impacts in EBD studies is that alternative scenarios may need to be specified for certain environmental risk factors, which can increase the complexity of the study [55]. It can also be difficult to select adequate indicators for estimating the burden of disease as they must balance feasibility with precision and validity [55].

Despite the challenges associated with conducting an EBD assessment, the outcomes can be used to inform policy and strategy in the health and environmental sectors, monitor health risks, and analyze the effectiveness of interventions [56]. The standardization of the EBD approach also allows for estimates from different studies and sources to be compared [56]. Additionally, it is possible to project exposures into the future and estimate trends in the environmental burden of disease, even if there is an extended time lag between exposure and the onset of disease [56]. Furthermore, EBD studies do not necessarily entail high costs and the calculations can be relatively simple once the exposure and health outcome data have been collected and compiled in a suitable format [56].

### 4.5. Lifecycle Methods

#### 4.5.1. Lifecycle Assessment

Lifecycle assessment (LCA) is an internationally regulated approach that aims to quantify multiple potential environmental impacts for a product, taking into account the whole lifecycle of the product from raw material extraction to final disposal [59,60,62,63]. It is frequently used by companies and policy-makers to aid in decision-making [62].

The LCA approach allows for the use of two different modeling principles for system analysis: attributional or consequential [59]. Attributional modeling is more widely used because it is easier to apply [59]. LCA studies can either be used to describe a single system or to compare different systems [59]. The LCA approach, as defined by the International Organization of Standardization (ISO 14040 and 14044), involves goal and scope definition, inventory assessment, impact assessment, and the interpretation of results [59].

LCA studies begin with the selection of system boundaries, which determine the processes that will be included [62]. An LCA study will ideally include all aspects of product development from cradle to grave [59,61]. However, many studies focus on specific stages of the production chain in order to simplify the analysis [59]. Many LCA studies investigating the impacts of milk production or the development of other animal-based food products choose to examine the potential impacts from cradle to farm-gate [59]. This scope allows for a better understanding of the potential environmental hot spots on farms [59].

Following the selection of system boundaries, researchers must then identify the functional unit. The functional unit is a quantifiable measure of the system and provides a reference for system inputs and outputs [60,62]. The selection of a functional unit is a controversial aspect of the LCA approach, especially when the method is applied to milk production [59]. The functional unit can be defined per product unit or per land area, and choosing to express environmental outputs per kilogram of product or per hectare of land can significantly alter the results of an LCA [59,61].

Next, an LCA study involves an inventory analysis phase, in which the resources consumed and the emissions to the environment are listed [63]. For an LCA of an agricultural production system, this includes both on-farm emissions and emissions related to the delivery of inputs to a farm [63]. Data collection for the inventory analysis phase is typically the most time-consuming part of an LCA study [59]. Most LCA studies of milk production include purchased feed, mineral fertilizers, fossil fuels, pesticides, replacement animals, transportation of inputs to the farm, and animal bedding materials [59]. Capital goods (for example, infrastructure and machinery) and veterinary drugs are rarely included in these studies due to a lack of data and heterogeneity between farms [59]. The handling of coproducts, such as beef on a dairy farm, is also an unresolved issue for the LCA approach [59]. Additionally, several emerging issues for agricultural production systems are not typically incorporated into LCA studies, such as biodiversity loss, land use change, and water consumption [59].

Following the inventory analysis phase, the potential environmental impacts are calculated based on characterization models that describe the environmental mechanism that links inventory data to an indicator [59,62,63]. Characterization models can be at either a regional or a global scale [59]. Impacts are calculated by multiplying the aggregate resources used and emissions produced by a characterization factor for each impact category to which it may contribute [63]. LCA studies can examine a number of different impacts, and some of the most commonly included are global warming potential, acidification potential, eutrophication potential, energy use, and land use [59]. Some studies have also examined ecotoxicity, ozone formation, human toxicity, ozone depletion, water depletion and abiotic depletion [59,61]. The LCA approach allows for an assessment of trade-offs and provides scope for those seeking to improve production practices [59]. One of the greatest strengths of the LCA approach is the system perspective; a broad perspective prevents the shifting of burdens from one environmental impact to another or from one stage of production to another [59]. LCA also has the potential to include social dimensions, although attempts to do so have been inconsistent [61].

#### 4.5.2. Lifecycle Costing

Lifecycle costing (LCC) is borne from the same methodological family as LCA and is the most common economic tool used jointly with LCA [61,64]. LCC allows for an assessment of all costs incurred throughout the entire production process, from resource extraction to final disposal [64]. LCC was developed in a management accounting context as a tool for ranking investment alternatives, with the primary goal of identifying the main cost factors on which business management should focus in order to optimize economic performance [64]. LCC is not standardized like LCA, but recently different procedures and standards have been developed in an attempt to harmonize the method [64]. Many LCC approaches are based on cash flow models in which future costs are actualized to their present value [64]. Unlike many conventional cost analysis methods, LCC is able to capture hidden costs that are usually overlooked without examining the full lifecycle [61].

Despite the fact that both LCA and LCC are lifecycle methods, they can be difficult to integrate due to differences in purpose, system boundaries, flows accounting, and timeframe [64]. LCA considers all processes connected to the physical lifecycle of a product from a multiple stakeholder perspective, while LCC considers all activities causing cost and benefit monetary flows during a product’s lifetime from a single stakeholder perspective [61,64]. LCC can be integrated with LCA through the creation of a common database, using the same functional unit and system boundaries, and assessing physical flows in monetary terms [64].

### 4.6. Integrated Assessment Models

Integrated assessment modeling was developed in an effort to capture complex multi-scale or multi-dimensional problems [68]. Integrated assessment models (IAM) are mathematical computer models based on explicit assumptions about how a modeled system behaves [66]. IAM incorporate a number of different sub-models or meta-models, often from different fields of study, which represent different components and organizational levels of a complex system into a single framework that allows for the transdisciplinary assessment of environmental and socioeconomic factors and impacts [66,68]. IAM typically attempt to quantify cause-effect relationships for a given problem, to the extent possible, as well as the cross-linkages and interactions between different factors [66]. IAM studies are usually undertaken to provide useful information to decision-makers and they bring together a number of different methods, tools, and research styles that would not typically be included in a study of the same issue within a single research discipline [66]. Furthermore, the outputs of IAM can often be linked with other approaches, such as HRA, or serve as complementary analyses [70]. For example, IAMs have frequently been applied to assessments of the impacts related to air pollution and climate change [66,70].

One strength of integrated assessment modeling is that it can be conducted at various spatial and temporal scales, which can allow for time-dependent analysis of impacts and the examination of spatial changes over time [69]. Additionally, IAM can incorporate multiple dimensions, including environmental, social, and economic aspects [68,73]. IAMs are also useful for organizing knowledge about an issue, understanding uncertainties, identifying knowledge gaps, and informing decisions about potential impacts and options for response [66]. However, IAMs are not prescriptive and results usually have a high degree of uncertainty [66]. IAM can provide general insights and inform the debate about how to respond, but, like all models, IAMs are constrained by the quality of the underlying assumptions [66].

### 4.7. Trade-Off Analysis

Trade-off analysis (TOA) is a multidisciplinary method that typically links site-specific environmental process models with economic decision models in order to examine the trade-offs between economic and environmental indicators [75,76]. The steps for conducting a TOA usually include: (i) identification of critical dimensions (that is, sustainability indicators) by stakeholders and scientists; (ii) formulation of hypotheses regarding the relationships between sustainability indicators and the definition of trade-off curves; (iii) identification of policy and technical interventions that could shift the defined trade-off curves; (iv) quantitative simulation of sustainability indicators under predefined scenarios; and (v) communication of results [75]. Quantitative simulations can be carried out at various spatial and temporal scales for different processes as appropriate, but in order to provide useful information to policy-makers, the simulations must be carried out for a sample that is representative for relevant populations [75]. TOA studies can include human health effects and can be used to show the potential trade-offs between economic and environmental health outcomes [75]. However, this approach has traditionally only been used to examine environmental and economic outcomes, not health or social impacts [75].

A major criticism of the TOA method is that the practical relevance of the models is often too limited [74]. For example, TOA models may not sufficiently account for different stakeholder perspectives or the broader policy environment [74]. Additionally, many TOA studies fail to adequately integrate interdisciplinary content [74]. For example, optimization approaches are particularly useful for assessing interventions, but are limited in their ability to incorporate social or cultural factors [74]. Furthermore, many TOA studies do not realize the full potential of assessing impacts across different spatial scales or include an appropriate representation of uncertainty [74]. However, the utility of the TOA method could be improved by using a combination of approaches [74].

### 4.8. Economic Assessment

An economic assessment (EA) can enumerate the potential costs and benefits of a proposed policy, program, initiative, or intervention. The economic valuation of proposals is critical because in many cases the environmental and health impacts will not be fully considered in policy-making without economic estimates [83]. Some EA can be very technical, but there are a number of tools for rapid or participatory assessments that can be easier to use [83]. While there are a variety of different tools available for conducting an EA, most tools follow the same steps: identification, measurement, valuation, and comparison of costs [84]. EA can be carried out at the individual level, for an industry or production sector, or for different geographic regions [84]. The scale used will determine which costs and benefits will be included in an assessment [84].

#### 4.8.1. Economic Valuation Methods

Stated preference and revealed preference methods are approaches commonly used to estimate economic values [84]. Stated preference methods rely on survey data in which individuals’ responses to questions about hypothetical markets or choices convey information about preferences [84]. Two different methods have been used for the stated preference approach: contingent valuation and choice modeling [84]. The contingent valuation method uses a survey to ask individuals about their willingness to pay for a single specific change [84]. Choice modeling also relies on survey data, but the questionnaire will typically include a series of questions with two or more answer options; analysts can then see how respondents value different characteristics that define different experiences by varying the answer options presented in the survey [84]. This method permits the valuation of incremental changes in attributes and is most appropriate for projects or policies that affect the individual aspects of a resource [84]. Revealed preference methods infer values from individuals’ market choices regarding goods and services related to the ones being investigated [84].

Existing economic value estimates from previous studies can also be used to transpose monetary values estimated in one location to another location using the benefits transfer approach [84]. Additionally, the travel cost method can be used for estimating the economic value of goods or services that are difficult to determine [84]. This method uses the costs incurred in reaching a location where goods or services can be obtained as a proxy for the value of those goods or services [84]. However, this method can be data intensive, especially when it relies on individual or household level travel behaviors and associated costs [84]. The method also relies on the assumption that the good or service obtained was an important determinant of travel behavior [84].

Human well-being is an intangible concept that cannot directly be measured and therefore economists often use willingness to pay (WTP) as a general single-scale composite indicator in order to define economic value in terms of economic behavior in the context of supply and demand [84]. In other words, WTP is the maximum amount of goods, services, or money that an individual is willing to give up in order to obtain an outcome that increases their personal well-being or welfare [84].

#### 4.8.2. Measuring Costs

There are several different approaches that are commonly used to measure the costs of policies, actions, or interventions [84]. The engineering analysis approach estimates the cost of an action for each step involved with implementation, this approach is often the simplest to understand and use [84]. The cost survey approach attempts to measure the costs of an action through surveys of relevant stakeholders [84]. One strength of this approach is that it considers the actual costs to individuals in practice. However, this approach is reliant on the quality of the survey and the quality of the responses [84]. Econometric estimates can be made at individual, sectoral, national, and international levels [84]. This approach controls for other important variables, as well as effects on trade and other markets and sectors [84]. However, econometric estimates can be data-intensive and time-consuming depending on the sophistication of the model used [84].

#### 4.8.3. Measuring Benefits

There are two different approaches for estimating the economic value of potential health benefits: the damage function approach and the cost-of-illness approach [84]. The quantification or valuation of benefits associated with an action or intervention using the damage function approach involves the identification of economically meaningful health impacts, identification and estimation of the expected change in health effect from the action or from alternative scenarios, and the estimation of the change in incidence of the health effect in the exposed population [84]. This approach requires the estimation of the economic value of adverse health effects avoided, and this unit value is then multiplied by the reduced incidence in the population in order to derive the monetized benefits [84]. On the other hand, the cost-of-illness approach combines estimated health care costs and work loss to determine the economic value of health benefits associated with a policy, program, or project [84]. However, this approach does not include other social or economic costs and, thus, does not reflect the total impact of an action or intervention on human well-being [84].

Other benefits, beyond those directly related to health, should also be considered in a comprehensive assessment and may need to be included in an estimation of the Total Economic Value [84]. Benefit valuation methods all follow the same basic steps: (i) identification of goods and services; (ii) assessment of provision or target level compared with baseline; (iii) identification of populations that benefit from the goods or services or suffer a loss when they are degraded; (iv) identification of possible values attributed to goods and services by the groups of people affected; (v) selection of an appropriate economic valuation method; (vi) estimation of the economic value or the change in provision level of goods and services; (vii) quantification of market size or the total population of beneficiaries over which the economic value is aggregated, accounting for possible distance-decay effects; and (viii) estimation of the Total Economic Value [84].

#### 4.8.4. Economic Valuation Methods for an Intervention or Alternative Scenarios

Cost/Benefit Analysis (CBA) is a systematic assessment in which the benefits of an action are contrasted with the associated opportunity costs within a common framework [84]. CBA typically follows these steps: (i) definition of the objective; (ii) definition of the baseline scenario; (iii) definition of alternative options or scenarios; (iv) quantification of investment costs for each option as compared with the baseline; (v) identification and quantification of both the positive and negative effects of each option as compared with the baseline; (vi) calculation of present value of costs and benefits occurring at different points in time; (vii) calculation of the net present value or the cost/benefit ratio for each option; and (viii) implementation of a sensitivity analysis [84]. The implementation of a CBA is usually a multidisciplinary exercise that requires expertise from economists, policy-makers, and scientists [84]. It is generally preferred that all costs and benefits included in a CBA be quantified in monetary terms, but this is not always possible [84]. In such cases, non-monetized impacts can still be discussed qualitatively and accompany CBA results [84].

Cost-effectiveness Analysis (CEA) is used to identify the most cost-effective option for achieving a previously defined objective that cannot be measured in monetary terms (for example, certain health outcomes) [84]. CEA is useful in situations where potential benefits or alternative options cannot be reliably estimated [84]. However, CEA is limited by its inability to identify benefits or society’s willingness to pay for changes or improvements. CEA usually entails the following steps: (i) definition of the objective; (ii) determination of the extent to which the objective is met (that is, how much progress has been made towards the goal already?); (iii) identification of current and future impacts over a set timeframe; (iv) identification of measures to move from baseline to target situation; (v) assessment of effectiveness of each measure; (vi) assessment of the cost of each measure; (vii) ranking of the measures in terms of increasing unit costs; and (viii) selection of a measure [84]. There are a number of different tools and approaches that can be used to carry out a CEA, depending on the level of complexity and the scale [84].

## 5. Application of Assessment Methods to Agricultural Production Systems

### 5.1. Risk Assessment Methods

Health risk assessments can be helpful in forming conclusions about health hazards from agricultural sectors in an objective and measurable way [4]. The HRA approach has been applied to various agricultural production sectors and specifically to livestock production in the case of zoonotic diseases, including Q fever, bovine spongiform encephalopathy, as well as in the case of antimicrobial resistant foodborne pathogens [4,46]. The HRA approach has also been applied to environmental pollutants such as nitrates [37], heavy metals, and trace metals [38], as well as zoonotic airborne pathogens emitted following the application of dairy manure to agricultural fields [35]. For example, researchers in China recently assessed the potential health risks associated with trace element contamination of drinking water in six different agricultural and animal husbandry regions [38]. Tap water samples were collected from 180 households and the levels of seven trace elements were analyzed [38]. The risks from carcinogenic and non-carcinogenic pollutants were assessed separately [38]. The evaluation models of health risk assessment recommended by the U.S. Environmental Protection Agency were used to estimate the health risk for adults and children and the estimates were compared with China’s health standards for drinking water [38].

Cumulative risk assessment has become an increasingly common approach for assessing chemical hazards and is beginning to be used more frequently to quantifying the risk associated with nonchemical stressors (for example, radiation, biological, psychological, and so forth) [41,42,43]. The CRA approach has been regularly used to assess the cumulative risk of pesticides [42], as well as phthalates [43] and air pollutants (including pollutants generated from industrial agricultural operations) [42,43]. The CRA approach was used in the National-Scale Air Toxics Assessment, which attempted to estimate the cancer and non-cancer health effects of joint exposure to air toxics across the United States [42]. Specifically, the assessment considered 177 air toxics and used atmospheric dispersion models to estimate concentrations on the basis of national emissions inventory [42]. The concentrations were then linked to population exposure and health risks were estimated [42]. While the analysis managed to include multiple agents or stressors, it did not manage to capture synergistic or antagonistic effects [42].

### 5.2. Health Impact Assessment

By contrast, the HIA approach has not been applied to agricultural systems as frequently as the HRA or EIA approaches [46,47]. Agricultural and food programs and policies have frequently been subject to EIA, but fewer HIAs have been applied to agricultural sectors [46]. Furthermore, there are more examples of HIAs conducted for specific smaller scale projects than for national policies [46]. However, HIAs have been conducted to examine the impact of the federal farm bill in the United States (US) and the impacts of the European Union’s (EU) Common Agricultural Policy (CAP) [47].

The Canadian government has also published two HIAs of regional agricultural sectors in Quebec, one for hog production and another for apple production [46]. The two HIA studies focused on how to integrate health impacts into an EIA framework [46]. The Canadian HIA manual discusses incorporating social impact assessment, epidemiology, health evaluation, economic, and risk assessment methods into the HIA framework and uses a quantitative approach to assess known health risks, mainly focusing on environmental pollution [46]. The Republic of Slovenia has also conducted an HIA for the national agricultural sector [46]. The HIA was undertaken by the government to examine the potential impacts of the adoption of the EU’s CAP when the nation applied to join the EU [46]. The HIA of CAP in Slovenia followed six distinct phases: policy analysis, rapid appraisal workshops with a wide range of stakeholders, a review of the empirical evidence relevant to the agricultural policy, an analysis of national data for key indicators related to health, a report on the findings, and an evaluation of the process [46].

A number of different tools have been developed to facilitate HIA studies, although only a few tools are publicly available, and most were developed for use in Europe, which limits their utility for an assessment of agricultural systems elsewhere. For example, DYNAMO-HIA (Dynamic Modeling for HIA) is a partial microsimulation model that simulates risk factor histories and calculates disease probabilities based on the Markov model [45]. This tool models the real-life population; projects baseline and intervention scenarios over time; and includes data on certain health determinants, such as smoking, overweight or obesity status, alcohol consumption, and related diseases like ischemic heart disease, stroke, diabetes, chronic obstructive pulmonary disorder, and five forms of cancer [45]. The model handles mortality selection due to earlier mortality among those exposed to explicit risk factors [45]. Additionally, the model has a parameter estimation module that helps to reduce data input requirements and still provides a relatively rich output [45]. DYNAMO-HIA is publicly available and has a graphic user interface that does not require programming skills to operate [45]. However, the tool was developed for use in Europe and only provides coverage of data for member states of the EU [45].

Another example is the combined Integrated Assessment of Health Risks of Environmental Stressors in Europe and Health and Environment Integrated Methodology and Toolbox for Scenario Assessment (INTARESE/HEIMTSA). These tools resulted from two large integrated projects funded by the European Commission for the development and implementation of a coherent methodology for integrated environmental health impact assessment [45]. INTARESE/HEIMTSA tracks the different environmental health impacts of policies [45]. Specifically, the tool projects how policy changes could affect air pollution emissions and concentrations with subsequent changes to human exposure and health impacts [45]. Health impacts are then aggregated into either DALYs or monetary values [45]. The tool is publicly available, but it is not a plug and play computational system which increases the difficulty of use [45]. Furthermore, the tool only includes data for Europe [45](Fehr 2012) and would not be applicable to an assessment conducted in another region. There are also a number of other tools that have been designed for use in quantitative HIAs including Age-Related Morbidity and Death Analysis (ARMADA), Health Forecasting, the Impact Calculation Tool (ICT), Proportional Multi-State Life Table (MSLT), Population Health Modeling (POHEM), Prevent, and RIVM-CDM [45]. However, most of these tools are not publicly available [45].

### 5.3. Environmental Impact Assessment Methods

The EIA approach has been frequently applied to assessments of agricultural programs and policies around the world [46,47]. EIA was first established in the US in 1969, but was quickly adopted in many other countries [52]. For example, in South Korea, a government-mandated EIA system was instituted in 1977 and EIA studies are now required for most agricultural projects, although specific guidelines were not developed for the agricultural sector [52]. The EIA approach has also been adopted throughout Europe and the first EU directive on EIA was issued in 1985 and specifically aimed to protect the environment, although implementation differed across countries [49,50]. For example, Denmark adopted the directive in 1989 and farmers were obligated to provide data to county-level regulators who were then required to undertake EIA studies for proposed livestock projects [49,50]. The EIA approach was also readily adopted by international organizations like the United Nations’ Food and Agriculture Organization (FAO) for use in planning development projects [51].

### 5.4. Environmental Burden of Disease

With regards to the EBD approach, several nations have conducted burden of disease studies and most of these studies have focused on the national pattern of disease burdens [55]. National burden of disease studies help to identify the most important risk factors and allow for the examination of intervention options [55]. Additionally, several studies have been undertaken to estimate the disease burdens from different environmental risk factors at either global, national, or regional scales [55]. Existing studies have focused on air quality, lead, noise, environmentally mediated infectious diseases, traffic accidents, and other environmental risk factors [55]. However, to the study team’s knowledge, the EBD approach has not been specifically applied to an agricultural system [55].

While the EBD approach has not specifically been applied to an agricultural system, it has been used to estimate the burden of disease attributable to a number of different environmental risk factors. For example, an EBD study conducted in Nepal examined the burden of disease attributable to temperature and climate change [57]. For the study, daily data for climate-sensitive variables and hospitalizations were collected for the five year period from 2009 to 2014, exposure-response modeling was conducted, and the environmental burden of disease attributable to climate-sensitive variables was estimated [57]. Specifically, morbidity and mortality data for waterborne (for example, typhoid, cholera, Hepatitis A and E), vector-borne (for example, malaria, dengue), heart (for example, ischemic heart disease, heart attack, hypertension), and renal (for example, chronic kidney disease, urinary tract infection) diseases, as well as all-cause mortality were assembled [57]. A linear model with a log-link function was used for exposure-response modeling and attributable fractions were estimated using WHO guidelines [57]. Attributable burdens were calculated for both a baseline (1985–2014) and a future (2015–2045) scenario, taking account of the effects of climate change while assuming that the total burden remained the same for both scenarios [57].

### 5.5. Lifecycle Methods

The LCA approach is considered to be a key tool for the assessment of the environmental impacts of an agricultural system [59,61,62]. LCA has commonly been used to examine greenhouse gas emissions and can use carbon dioxide (CO_2_) equivalents to aggregate emissions of different gases along the supply chain [62]. LCA studies can also be used to compare impacts for different agricultural systems and products [60,61] and has frequently been used to examine the impacts of milk production [59,60]. A review of LCA studies of milk production found that most studies have been conducted in Europe, especially in France, Ireland, and Italy [59]. This concentration reflects the long-term focus on the environmental sustainability of the dairy sector in those regions [59]. The influence of different farm management practices has been evaluated in a number of different ways; some studies have compared a priori two different management systems, others have a posteriori considered a large number of farms in an effort to determine which farm characteristics are the most important, and some have focused on the economic impacts [59].

A number of attempts have been made to standardize the use of the LCA approach for assessing the impacts of livestock production and dairying. In 2016, the Livestock Environmental Assessment and Performance (LEAP) Partnership released the “Environmental Performance of Large Ruminant Supply Chains: Guidelines for Assessment,” which represents a recent attempt to standardize the use of LCA in the livestock sector [59]. The International Dairy Federation also published a guide that attempted to standardize LCA for the dairy sector in 2010, but few studies have adhered to these guidelines since they were published [59].

The LCC approach can be used as a decision support tool within an LCA of food products, but there are few examples of LCC being applied directly to a food product in the literature [61,64]. However, the LCC approach can be combined with a cash flow analysis in order to determine the profitability of agricultural systems through economic indicators [64] or potentially combined with LCA results in order to estimate the long-term externalities of agricultural production systems [61].

### 5.6. Integrated Assessment Models

While IAMs have frequently been applied to assessments of the impacts related to climate change [66], only in recent years have a number of models been developed for agricultural systems [68]. Specifically, a number of advanced models developed to investigate the impacts of climate change have been extended to examine agricultural productivity and potential human health impacts. For example, the Massachusetts Institute of Technology (MIT) developed an IAM called the MIT Integrated Global System Modeling (IGSM) Framework [71]. The main component of the framework is the MIT Emissions Predictions and Policy Analysis (EPPA) Model and the standard atmospheric component is two dimensional atmospheric model based on the Goddard Institute for Space Studies’ General Circulation Model for climate, coupled with an ocean model with the treatment of heat and carbon flows into the deep ocean [71]. A number of other models can be linked within the framework, including a reduced-form urban chemical model that can be used to better represent smaller scale urban chemical processes that influence air chemistry and climate [71]. The Global Land System component links the US National Center for Atmospheric Research’s Community and Land Model; the US Marine Biological Laboratory’s Terrestrial Ecosystem Model that simulates carbon dioxide fluxes and the storage of carbon and nitrogen in vegetation and soils; and the Natural Emissions Model that simulates methane and nitrous oxide fluxes [71]. The framework then links econometric decisions regarding the spatial pattern of land use and land use conversion to examine the impacts of land use change and greenhouse gas emissions [71].

While the MIT IGSM framework was primarily developed to examine the environmental impacts of climate change, the framework can be extended to investigate the impacts of climate change on human health and provide economic estimates for health impacts using an economic accounting approach [71]. Extending the model involves the valuation of non-wage time (for example, leisure) and the inclusion of health services produced at the household level to capture the economic effects of morbidity and mortality from acute exposures [71]. Specifically, the model considers both market and non-market effects [71]. For example, the death or illness of a person in the labor force or expenditures on medical services are market effects, while death and illness also involve the loss of non-paid time or productivity, which is a non-market impact [71]. The framework can also be used to examine the impacts of climate change on agriculture by disaggregating the agricultural sector within the EPPA model, which allows for the simulation of economic effects of changes in production yield on regional economies and trade [71].

Another example is the Integrated Model to Assess the Global Environment (IMAGE), which was developed to examine the long-term dynamics of global change and includes a number of different biophysical modules for ecosystems, agricultural production, land use, and environmental effects [67,72]. IMAGE has been used in a number of different studies to assess potential environmental impacts of agricultural systems [67,72]. For example, one study linked two different economic models to the IMAGE integrated assessment model to examine different alternatives for the reduction of the environmental impacts of agriculture in the EU and globally [72]. Specifically, the study examined the following scenarios: (i) baseline; (ii) changing diets in the European Union to be consistent with the World Health Organization’s recommendations by 2020; (iii) reducing global food waste from 20% to 5% by 2020; (iv) increasing global crop yields to 40% higher than baseline; and (v) increasing global livestock feed efficiency to 15% higher than the baseline by 2020 [72]. The study used two different global economic models: IMPACT and LEITAP, a partial equilibrium model and a static, applied computable general equilibrium model, respectively [72]. The two models were used to calculate the regional production of different agricultural commodities, which were then entered into the IMAGE integrated assessment model to calculate the potential environmental impacts, including land use, greenhouse gas emissions, and climate change under each scenario [72]. The use of the IMAGE integrated assessment model coupled with two different economic models allowed for the study to account for feedback systems within the global agricultural sector that had previously been overlooked [72].

Generally, country-level IAMs can allow for time-dependent assessment of climate change and the examination of spatial changes over time in regions of economic importance for agricultural production [69]. A typical characteristic of agricultural systems is that the components are often shaped by both environmental and socioeconomic factors and one of the strengths of integrated assessment modeling is that it can incorporate those dimensions ([68,73]. However, some IAMs are heavily biased towards a single dimension and are imbalanced with the degree of quantification [73]. For example, social factors and impacts (for example, employment), income distribution, and the quality of life for farmers are not generally well represented [73].

### 5.7. Trade-Off Analysis

The TOA approach has frequently been used in the agricultural sector [74,76]. In agriculture, trade-offs can arise at many different levels, from crop or animal to field, farm, or landscape [74]. Farmers face trade-offs between maximizing production in the short-term and ensuring sustainable production in the long-term [74]. At the landscape level, there are trade-offs between different land uses [74]. Trade-offs can also occur between different environmental, social, economic, and cultural objectives; across different spatial and temporal scales; and between different stakeholder groups [74]. TOA models have frequently been used to examine agricultural systems and mathematical programming is possibly the most widely used TOA approach for assessing land use options [74]. Mathematical programming is an optimization approach that can be used to find the best possible trade-off using multi-criteria analysis [74]. TOA studies of agricultural systems often incorporate local scale crop models to assess the land quality and economic models to simulate land management decisions [76]. Land management decisions can then be used as inputs in an environmental process model that simulates environmental outcomes [76]. Economic and environmental outcomes can then be aggregated to a regional level and used to develop indicators that can be used to examine trade-offs [76]. This approach can be used to estimate the effect of different policy scenarios [76].

### 5.8. Economic Assessment

Economic assessment methods have been applied to agricultural systems in a number of different ways in order to value both environmental and health impacts. Economic valuation approaches have been extensively applied to the issue of pesticide risks [77,79,82]. For example, an empirical study in Northern Italy estimated the economic value of reducing the environmental and health impacts of agricultural pesticide use through a Choice Experiment approach [82]. The study specifically focused on the reduction in farmland biodiversity, groundwater contamination, and acute human illnesses and relied on stated preference non-market valuation techniques to infer people’s preferences (that is, willingness to pay) regarding decreases in pesticide impacts and corresponding increases in grocery expenditures [82]. Another study, conducted in Canada, combined a biophysical risk assessment approach with a contingent valuation survey on consumers’ willingness to pay for reductions in pesticide risk [77].

Economic assessment methods have also been used to estimate the external environmental and health costs associated with agricultural production systems in parts of Europe and the United States [80,81]. For example, an assessment of externalities in the United Kingdom used a framework of seven different cost categories, including damage to water, damage to soil, damage to air, damage to natural capital, damage to human health from pesticides, damage to human health from nitrate, and damage to human health from pathogens [80]. The study estimated ranges for two different types of damage costs: (i) treatment and prevention costs and (ii) administrative and monitoring costs [80]. The study found that significant costs arose from the contamination of drinking water with pesticides, *Cryptosporidium spp.*, phosphate and soil; damage to wildlife, habitats, hedgerows, and drystone walls; soil erosion and organic carbon losses; food poisoning; and bovine spongiform encephalopathy [80]. Another study conducted in the UK combined the LCA approach with economic valuation approaches including the willingness to pay, revealed preference values, and econometric estimates in order to assess the environmental, economic, and social impacts of the livestock sector [78]. Specifically, the study examined a series of future scenarios to determine the potential effects on ecosystem services including food production and provisioning, environmental regulation, and cultural benefits such as recreation [78]. Altogether, there are numerous ways to apply different EA methods to inform and support decision-making in the agricultural sector.

Box 2 **The applicability of methods to an assessment of the New Zealand dairy sector.**

Ideally, a method for assessing the potential environmental health impacts associated with dairying in NZ should also include consideration of social and economic dimensions. The method would be applied at a national scale in order to inform national level policy, but should also allow for the examination of global, regional, or local level impacts where relevant. Furthermore, the method should also allow for the examination of potential future impacts and the analysis of different policy scenarios. It should also generate outputs in a format suitable for presentation to government officials and which support policy change decisions.

Based on this broad review and comparison (Table 3) of eight different approaches, health impact assessment (HIA) may be the most appropriate method for use in an assessment of the NZ dairy sector. The environmental burden of disease (EBD) and the integrated assessment modeling (IAM) approaches may also be useful in this context, although to the authors’ knowledge the EBD approach has not yet been applied to an assessment of the health impacts of an agricultural sector and the IAM approach would only be suitable if health impacts could be adequately incorporated.

In many ways, the HIA, EBD, and IAM approaches share similar attributes. All three approaches have been used to provide information to policy-makers at various levels of government and each could be used to inform either sector-specific or government-wide regulators. The three approaches can all be applied at a national scale, but can also be used to examine local, regional, or global impacts. Additionally, these methods can all account for both short- and long-term effects. However, the approaches differ substantially in other ways.

HIA is the only method that covers environmental, health, social, and economic dimensions. Additionally, the method was specifically designed to assess the impacts of a proposed policy, while EBD and IAM were developed with different aims (Table 2). Unfortunately, the HIA approach does not typically support the thorough assessment of alternative scenarios, but generally seems to be the most appropriate method for an assessment of the potential impacts of the NZ dairy sector.

The EBD approach may also be an appropriate assessment method, but EBD does not typically consider social or economic dimensions. The method has been simplified and detailed guides have been developed to reduce the time and resources required to complete a national or subnational study [56], but there is a trade-off between the accuracy of estimates and the level of effort required for data collection [55]. In contrast to the HIA approach, EBD studies can support the exploration of alternative scenarios. Generally, if the EBD approach was modified or expanded to account for social and economic dimensions, then the method might prove useful for assessing the impacts of the dairy sector in NZ.

IAM may also prove a powerful approach for an environmental health assessment of an agricultural system, but only if the method could be modified to consistently and comprehensively consider health impacts. Generally, IAM studies only tend to incorporate environmental, economic, and social dimensions. IAM is a promising approach for an assessment of a complex system, like the NZ dairy sector, because it was specifically developed in an effort to capture complex multi-scale or multi-dimensional problems. Like the EBD approach, IAM supports the consideration of alternative future scenarios. However, similar to HIA, the outputs can be highly technical and IAM studies can require significant time and resources to complete. Overall, the IAM approach shows promise for use in assessing multiple dimensions of complex systems.

In NZ, certain environmental health impacts associated with dairy farming merit further investigation using an approach that accounts for cross-sectoral drivers and impacts, such as health impact assessment, environmental burden of disease, or integrated assessment modeling.

## 6. Conclusions

A review of eight different environmental health assessment methods reveals the diversity of the aims, dimensions, processes, and concepts involved. With the exception of the environmental burden of disease approach, all of the reviewed methods have been applied to agricultural systems. The suitability of an assessment method for a given purpose will depend on a variety of factors, but the approach chosen should reflect the complexity of agricultural systems and the multitude of potential environmental, economic, and human health effects. Generally, approaches that include a single dimension produce an incomplete picture and the inclusion of economic and social benefits, along with health and environmental dimensions, can provide a more holistic view of an agricultural system. This review can provide a starting point for selecting an existing method or designing a new method for assessing the environmental health impacts of an agricultural system.

## Figures and Tables

**Table 1:** The identified methods applicable to assessing the environmental health impacts of an agricultural system and number of references included in the review.

Method	Number of References Included	References
(1a) Health risk assessment (HRA)	7	[4,34,35,36,37,38,39]
(1b) Cumulative risk assessment (CRA)	4	[40,41,42,43]
(2) Health impact assessment (HIA)	5	[44,45,46,47,48]
(3a) Environmental impact assessment (EIA)	6	[15,49,50,51,52,53]
(3b) Environmental health impact assessment (EHIA)	1	[53]
(3c) Strategic environmental assessment (SEA)	2	[53,54]
(4) Environmental burden of disease (EBD)	4	[55,56,57,58]
(5a) Lifecycle assessment (LCA)	6	[15,59,60,61,62,63]
(5b) Lifecycle costing (LCC)	3	[61,64,65]
(6) Integrated assessment modeling (IAM)	8	[66,67,68,69,70,71,72,73]
(7) Trade-off analysis (TOA)	3	[74,75,76]
(8) Economic assessment (EA)	8	[77,78,79,80,81,82,83,84]

**Table 2:** The characteristics of methods applicable to assessing the environmental health impacts of an agricultural system.

Method	Aim of Method	Development
(1a) HRA	To estimate the probability of adverse health effects in humans who may be exposed to a specific hazard	Generally credited to Dr. Lewis C. Robbins who created the first health hazard charts [36]; substantial involvement of Canadian and United States (US) government agencies in the 1970s and 1980s led to further development of HRA programs and tools [36]
(1b) CRA	To analyze, characterize, and possibly quantify the combined risks to health or the environment from multiple agents of stressors	CRA was developed out of the HRA approach when the United States Environmental Protection Agency (EPA) was directed by the Food Quality Protection Act (FQPA) of 1996 to consider the cumulative effects of chemical exposures that occur simultaneously [40]; the first formal step towards developing guidelines for CRA was taken in 1997, when the EPA Science Policy Council (SPC) issued guidance on planning and scoping for CRA [40,41]; the EPA SPC subsequently tasked the Risk Assessment Forum with drafting an agency-wide framework for the CRA process in the early 2000s [40]
(2) HIA	To assess the potential health impacts of a proposed policy, program, project, or plan; HIA is a predictive tool to support decisions in policy-making; the ultimate goal is to maximize health gains and reduce health inequities	The HIA field grew out of environmental impact assessment and gained legitimacy following the publication of the Gothenburg Consensus Paper in 1999 by the World Health Organization (WHO), which outlined the main concepts and suggested approaches for conducting HIA [44]; numerous approaches and tools have been developed internationally [45]
(3a) EIA	To assess the potential environmental impacts of a proposed policy, program, project, or plan	EIA was formally developed in the US in 1969 with the enactment of the National Environmental Policy Act [52] and in the European Union (EU) in 1985 with the issuance of EU Directive 85/337/EEC [49,50]; the EU Directive indicated that EIA was intended to be used as a preventative regulatory tool and has since been used all over the world [49,50]
(3b) EHIA	To assess the potential environmental health impacts of a proposed policy, program, project, or plan	Developed out of EIA, but includes a health component in the appraisal process; the analysis of health impacts is not as focused as with HIA [53]
(3c) SEA	To assess the potential environmental and health impacts of a proposed policy, program, project, or plan	Developed out of EIA, but places emphasis on human health impacts in addition to environmental impacts and is usually undertaken earlier in the decision-making process for proposals; the SEA method provides the opportunity for health to be thoroughly considered within an environmental assessment framework [53]
(4) EBD	To provide a quantitative estimate of the health impact (usually measured in disability-adjusted life years/DALYs) attributable to an environmental exposure	The first Global Burden of Disease (GBD) study was published in the early 1990s in a report commissioned by the World Bank and was conducted in a collaboration between the WHO and Harvard University Dept. of Public Health [58]; the EBD method was developed out of the GBD approach in the late 1990s and early 2000s [55]
(5a) LCA	To assess the environmental impacts associated with all of the stages of a product’s lifecycle	First developed as a tool for manufacturing operations [62] and was later standardized by International Organization for Standardization in 2006 [59]
(5b) LCC	To assess the monetary costs and benefits associated with all of the stages of a product’s lifecycle	Developed out of the LCA methodology in a management accounting context as a tool for ranking investments [64,65]; adopted by US military in the mid-1960s and then applied to building assets [65]
(6) IAM	To assess the complex interrelationships between natural and social factors that underlie environmental problems, such as climate change	The first major integrated assessment for an environmental issue may have been the Climatic Impact Assessment Program, which investigated potential atmospheric impacts of the proposed American supersonic transport aircraft in the early 1970s [66]; IAM was later used by the US Department of Energy in the late 1970s for a program to examine the potential impacts of climate change, and by the International Institute for Applied Systems Analysis in the early 1980s to model acid rain in Europe [66]. Recently, IAM has been used for global climate change and air pollution assessments and IAM results subsequently provided the basis for Intergovernmental Panel on Climate Change (IPCC) assessment reports [70].
(7) TOA	To quantify the trade-offs within agricultural systems between environmental, economic, and other objectives	The concept of analyzing trade-offs is fundamental to economics, but TOA process was first proposed for use in providing quantitative information to support policy decision-making about agricultural production systems in the late 1990s [75]
(8) EA	To enumerate the potential costs and value potential benefits associated with a proposed policy, program or project	There is not a harmonized methodology for estimating economic costs and benefits for the environmental health field; a number of different approaches have been used [83]

**Table 3:** The comparison of environmental health assessment method attributes **.

Method	Dimensions Typically Emphasized				Types of Decisions Typically Supported		Temporal Scale		Spatial Scale				Time to Complete	Ease of Use		Consideration of Uncertainty
	Economic	Environmental	Health	Social	Assessing Impacts of Processes, Products, Pollutants	Assessing Policies, Programs, Projects, Plans	Short-Term Effects	Long-Term Effects	Local	Regional	National	Global/International		Data Requirement	Technical Expertise	
HRA	+	+	+++	+	+++	+	+++	+++	+++	+++	++	+	+++	++	++	+++
CRA	+	++	+++	+	+++	++	+++	+++	+++	+++	++	+	+++	+++	+++	+++
HIA	++	+++	+++	+++	+	+++	+++	+++	++	+++	+++	+++	+++	+++	+++	+++
EIA	+	+++	+	+	+	+++	+++	+++	+++	+++	+++	+++	+++	+++	+++	++
EHIA	+	+++	++	+	+	+++	+++	+++	+++	+++	+++	+++	++	+++	+++	++
SEA	+	+++	+++	+	+	+++	+++	+++	+++	+++	+++	+++	++	+++	+++	++
EBD	+	+++	+++	+	+++	+	+++	+++	+++	+++	+++	+++	++	++	++	++
LCA	+	+++	++	++	+++	++	+	+	+	++	++	++	++	+++	++	+++
LCC	+++	+	+	+	+++	++	+++	+++	+	+	+	+	+++	+++	++	++
IAM	+++	+++	+	+++	++	+++	+++	+++	+++	+++	+++	+++	+++	+++	+++	+
TOA	+++	+++	++	+	++	++	+++	+++	+++	+++	++	+	+++	++	++	++
EA	+++	+	+	+	++	++	+++	+++	+++	+++	+++	+++	++	+++	++	++

Symbols indicate the extent for each assessment method: +++ Major degree, ++ Moderate degree, + Minor degree or not at all. ** Non-quantitative comparison of method attributes is subjective and based on a limited number of examples and articles.

## Appendix A

### Appendix A.1. Medline Search Strategy (Conducted 11 July 2017, Retrieved 173 Articles)

1. exp *Environmental Exposure/ or exp *Environmental Pollution/ or exp *Environment/

2. an.fs. [Analysis]

3. 1 and 2

4. ((environment* or health or ecolog*) adj2 (impact* or effect* or pollut* or toxic* or influenc* or consequence* or outcome* or ramification* or repercussion* or negative* or positive* or risk* or consequence*)).ti.

5. 3 or 4

6. exp *animal husbandry/ or dairying/ or Livestock/

7. (livestock or “live-stock” or dairying or (dairy adj1 (farm* or production or industr* or intensive* or herd* or land*))).ti.

8. 6 or 7

9. 5 and 8

10. *Risk Assessment/mt or exp *Data Collection/mt [Methods]

11. (assess* or measur* or evaluat* or calculate or quantify or test* or analys*).ti.

12. 10 or 11

13. 9 and 12

14. limit 13 to english language

### Appendix A.2. Scopus Search Strategy (Conducted 11 July 2017, Retrieved 437 Articles)

((TITLE-ABS-KEY (“environmental exposure” OR environment*) OR TITLE (environment* OR health OR ecolog* W/2 (impact* OR effect* OR pollut* OR toxic* OR influenc* OR consequence* OR outcome* OR ramification* OR repercussion* OR negative* OR positive* OR risk* OR hazard*))))

AND

(TITLE (“animal husbandry” OR “livestock production” OR livestock OR dairying OR (dairy* W/1 (farm* OR production OR industr* OR intensive* OR herd* OR land*))))

AND

((TITLE-ABS-KEY (“risk assessment” OR “impact assessment”) OR TITLE (assess* OR measur* OR evaluat* OR calculate OR quantify OR test* OR analy*)))

AND

(LIMIT-TO (DOCTYPE, “ar”) OR LIMIT-TO (DOCTYPE, “re”) OR LIMIT-TO (DOCTYPE, “ip”))

AND

(LIMIT-TO (SRCTYPE, “j”)) AND (LIMIT-TO (LANGUAGE, “English”)) AND (EXCLUDE (SUBJAREA, “VETE”))

### Appendix A.3. Web of Science Search Strategy (Conducted 12 July 2017, Retrieved 697 Articles)

1. TITLE: ((environment* or health or ecology*) NEAR/2 (impact* or effect* or pollut* or toxic* or influenc* or consequence* or outcome* or ramification* or repercussion* or risk* or benefit* or positive* or negative*))

Indexes=SCI-EXPANDED, SSCI, A&HCI, CPCI-S, CPCI-SSH, ESCI, CCR-EXPANDED, IC Timespan=All years

2. TOPIC: (“animal husbandry” or dairying or livestock or agriculture*)

Indexes=SCI-EXPANDED, SSCI, A&HCI, CPCI-S, CPCI-SSH, ESCI, CCR-EXPANDED, IC Timespan=All years

3. TITLE: (livestock or dairy* or agricultur* NEAR/1 (farm* or production or industr* or intensi* or herd* or land*))

Indexes=SCI-EXPANDED, SSCI, A&HCI, CPCI-S, CPCI-SSH, ESCI, CCR-EXPANDED, IC Timespan=All years

4. TITLE: (assess* or measure* or evaluat* or calculate* or quantif* or analy*)

Indexes=SCI-EXPANDED, SSCI, A&HCI, CPCI-S, CPCI-SSH, ESCI, CCR-EXPANDED, IC Timespan=All years

5. #3 OR #2

Indexes=SCI-EXPANDED, SSCI, A&HCI, CPCI-S, CPCI-SSH, ESCI, CCR-EXPANDED, IC Timespan=All years

6. #5 AND #4 AND #1

Indexes=SCI-EXPANDED, SSCI, A&HCI, CPCI-S, CPCI-SSH, ESCI, CCR-EXPANDED, IC Timespan=All years

7. #5 AND #4 AND #1 **Refined by: DOCUMENT TYPES:** (ARTICLE)

Indexes=SCI-EXPANDED, SSCI, A&HCI, CPCI-S, CPCI-SSH, ESCI, CCR-EXPANDED, IC Timespan=All years

8. #5 AND #4 AND #1 **Refined by: DOCUMENT TYPES:** (ARTICLE) AND **LANGUAGES: (ENGLISH)**

Indexes=SCI-EXPANDED, SSCI, A&HCI, CPCI-S, CPCI-SSH, ESCI, CCR-EXPANDED, IC Timespan=All years

### Appendix A.4. GreenLINE Search Strategy (Conducted 12 July 2017, Retrieved 130 Articles)

1. TI (livestock or “live-stock”or dairying or “animal husbandry” or (dairy N1 (farm* or production or industr* or intensive* or herd* or land*))) OR SU (livestock or “live-stock”or dairying or “animal husbandry” or (dairy N1 (farm* or production or industr* or intensive* or herd* or land*))) OR KW (livestock or “live-stock”or dairying or “animal husbandry” or (dairy N1 (farm* or production or industr* or intensive* or herd* or land*)))

2. TI ((environment* or health or ecology*) N2 (indicat* or impact* or effect* or pollut* or toxic* or influenc* or consequence* or outcome* or ramification* or repercussion* or negative* or positive* or risk*)) OR SU ((environment* or health or ecology*) N2 (indicat* or impact* or effect* or pollut* or toxic* or influenc* or consequence* or outcome* or ramification* or repercussion* or negative* or positive* or risk*)) OR KW ((environment* or health or ecology*) N2 (indicat* or impact* or effect* or pollut* or toxic* or influenc* or consequence* or outcome* or ramification* or repercussion* or negative* or positive* or risk*))

3. S1 AND S2

4. TI (assess* or measure* or evaluat* or calculate or quantify or test* or analys* or estimate* or determin* or indicat*) OR SU (assess* or measure* or evaluat* or calculate or quantify or test* or analys* or estimate* or determin* or indicat*) OR KW (assess* or measure* or evaluat* or calculate or quantify or test* or analys* or estimate* or determin* or indicat*)

5. S3 AND S4

6. S3 AND S4, Narrow by Language: - English
